# A Mobile Health Approach for Improving Outcomes in Suicide Prevention (SafePlan)

**DOI:** 10.2196/17481

**Published:** 2020-07-30

**Authors:** Conor O'Grady, Ruth Melia, John Bogue, Mary O'Sullivan, Karen Young, Jim Duggan

**Affiliations:** 1 School of Computer Science National University of Ireland Galway Galway Ireland; 2 Health Service Executive Ennis Ireland; 3 School of Psychology National University of Ireland Galway Galway Ireland; 4 HSE Community Healthcare West Galway Ireland

**Keywords:** mobile apps, suicide, mHealth

## Abstract

**Background:**

Suicide is a prominent cause of death worldwide, particularly among young people. It was the second leading cause of death among those aged 15-29 years globally in 2016. Treatment for patients with suicidal thoughts or behaviors often includes face-to-face psychological therapy with a mental health professional. These forms of interventions may involve maintaining and updating paper-based reports or worksheets in between sessions. Mobile technology can offer a way to support the implementation of evidence-based psychological techniques and the acquisition of protective coping skills.

**Objective:**

This study aims to develop a mobile app to facilitate service users’ access to mental health support and safety planning. This process involved eliciting expert input from clinicians who are actively engaged in the provision of mental health care.

**Methods:**

A survey was distributed to targeted health care professionals to determine what features should be prioritized in a new mobile app relating to suicide prevention. On the basis of the survey results, a clinical design group, comprising 6 members with experience in fields such as mobile health (mHealth), clinical psychology, and suicide prevention, was established. This group was supplemented with further input from additional clinicians who provided feedback over three focus group sessions. The sessions were centered on refining existing app components and evaluating new feature requests. This process was iterated through regular feedback until agreement was reached on the overall app design and functionality.

**Results:**

A fully functional mobile app, known as the SafePlan app, was developed and tested with the input of clinicians through an iterative design process. The app’s core function is to provide an interactive safety plan to support users with suicidal thoughts or behaviors as an adjunct to face-to-face therapy. A diary component that facilitates the generalization of skills learned through dialectical behavior therapy was also implemented. Usability testing was carried out on the final prototype by students from a local secondary school, who are representative of the target user population in both age and technology experience. The students were asked to complete a system usability survey (SUS) at the end of this session. The mean overall SUS rating was 71.85 (SD 1.38).

**Conclusions:**

The participatory process involving key stakeholders (clinicians, psychologists, and information technology specialists) has resulted in the creation of an mHealth intervention technology that has the potential to increase accessibility to this type of mental health service for the target population. The app has gone through the initial testing phase, and the relevant recommendations have been implemented, and it is now ready for trialing with both clinicians and their patients.

## Introduction

According to the Global Observatory for eHealth, mobile health (mHealth) is defined as “medical and public health practice supported by mobile devices, such as mobile phones, patient monitoring devices, personal digital assistants (PDAs), and other wireless devices” [1]. The World Health Organization (WHO) states that “mHealth involves the use and capitalization on a mobile phone’s core utility of voice and short messaging service as well as more complex functionalities and applications including general packet radio service, third and fourth generation mobile telecommunications (3G and 4G systems), global positioning system, and Bluetooth technology” [1].

Mobile devices have the potential to deliver evidence-based interventions with greater customization to the individual and at the time when the intervention is required. mHealth programs use mobile technology for a variety of functions, ranging from data collection tools for health care professionals and clinical decision support systems to support health behavior change by patients in the community [2].

The WHO reported that an estimated 800,000 people die by suicide each year globally, constituting a major public health concern. Suicide is the second leading cause of death in young people aged 15 to 24 years [3]. In Ireland, provisional figures indicate that 352 individuals died by suicide in 2018 [4]. Approximately one-third of people who died by suicide had been in contact with mental health care in the year before their death, and approximately 1 in 5 people had contact with a professional in the month before death [5]. Therefore, self-harm and suicide attempts (SAs) represent a pivotal opportunity for intervention. Suicide prevention guidelines recommend safety planning alongside treatment as usual for patients at risk for mental health care [6]. However, safety planning is often not implemented in practice [7], and the feasibility of carrying paper-based plans may be limited, given the transient nature of suicide risk states [8]. Stigma and geographical isolation are two major barriers to help seeking for individuals at risk of suicide [9]. Advancements in mHealth technology could lower these main barriers by directing individuals at risk of suicide, who would not otherwise seek help, to appropriate evidence-based, web-based apps or traditional mental health interventions [10]. Research has indicated the probable benefits of internet-based suicide prevention methods [11]. Furthermore, a survey completed by psychiatric outpatients outlined that 69 of 98 participants (70%) expressed an interest in using a mobile app to track their mental health on a daily basis [12]. Given the established challenge of suicide in young people [3], there was an identified need to design and develop an app for use by 17- to 35-year-old secondary-level mental health service users (adult mental health or child and adolescent mental health). Specifically, those who present with suicidal ideation, nonsuicidal self-injury, or a history of a previous SA. A new mobile app, which is titled SafePlan and developed by the National University of Ireland Galway, was designed to be used as an adjunct to therapy for this at-risk group.

Before the design and development process of SafePlan, a review of existing mobile apps (N=5) providing support in the area of mental health and suicide prevention was conducted (Table 1). These apps were selected based on their subject and overall app store ranking. The review identified a functionality gap whereby none of these apps were combining the capabilities of safety plans, diaries, and other therapeutic intervention worksheets in one. The reviewed apps tended to focus on only one of these support methods, and the majority of them failed to provide any reporting tools for sharing user data.

**Table 1:** Review of existing apps in the area of mental health and suicide prevention.

App names	Developers	Platforms (iOS/Android)	Safety plan	Diary	Dialectical behavior therapy	Sharing
Suicide Safety Plan [13]	MoodTools	Both	✓^a^	x^b^	x	x
Safety Plan [14]	Blue Bird Technologies	Android	✓	x	x	x
MYPLAN-Your safety plan [15]	Minplan, Denmark	Android	✓	x	x	x
Dbt112 [16]	annadroiddev@gmail.com	Android	x	✓	✓	✓
DBT^c^ Travel Guide [17]	dialexisadvies.nl	Both	x	x	✓	✓

^a^✓: contained this functionality.

^b^x: did not contain this functionality.

^c^DBT: dialectical behavior therapy.

Dialectical behavior therapy (DBT) is an evidence-based program aimed at helping people with ongoing difficulties in managing intense emotions. It is used in the treatment of mood disorders, suicidal ideation, and changing behavioral patterns, such as self-harm and substance abuse [18]. In clinical practice, both safety planning and DBT interventions may be combined to provide a tailored approach to suicide prevention treatment. There are many apps currently on the market that specialize in one of these methods, which require a user to download and maintain two unrelated apps if they wish to support these treatments electronically. The lack of a centralized resource for safety planning and DBT supports was a highly motivational factor in the development of a new app that would combine these prevention techniques. Early design meetings also identified that this approach should support additional intervention methods in future versions of the app by using a modular and flexible design architecture. The user could then enable or disable these options within the app’s settings menu. This idea of intervention flexibility was well received by all members of the design team as they felt it had the potential to open the app to a much wider user base.

The proposed new app should have the added advantage of being co-designed with professional clinicians to support best practice patient-professional interaction based on valid data. Traditionally, both safety planning and DBT treatments involve patients maintaining and updating paper-based reports or worksheets between sessions. Mobile technology can provide an opportunity to streamline this process by eliminating the need for these paper-based resources and hence reduce human error and potential loss of material. It also helps to facilitate the recording of real-time data in a timely manner. The migration of these records to a mobile app could have a major impact on the accuracy and completeness of the data being collected [19].

Finally, the lack of any sharing functionality within existing safety planning apps summarized in Table 1, highlighted a further key requirement for the SafePlan app. This was particularly relevant because of the app’s intended use envisaged as being an adjunct to therapy, that is, the information within the app should play a key role in patient-professional sessions. The ability to share data with a clinician or trusted helper was a core focus for the early stages of design. Users taking control of their own health information is a growing trend in digital and participatory health care [20,21]. A number of methods were analyzed for the extraction of app data in a user-friendly format, and a consensus was reached whereby a user could wirelessly print a safety plan or DBT report directly from their phone through the app’s *My Reports* screen. This option was chosen primarily for its ease of use; however, it also plays an important role in the overall security and privacy of the app. Passing data to web-based portals or cloud accounts was considered too risky an approach based on the confidential nature of the app’s data. This is also the reason why it was decided to store all the app’s user-entered data locally on the user’s device as opposed to an external server. In summary, the aim of this study was to investigate whether mobile technology can offer a way to support the implementation safety planning approaches and therefore support the acquisition of protective coping skills for patients at risk.

## Methods

### Design Process

The SafePlan app was built using an agile software development approach [22]. The agile methodology is an incremental model of app development where requirements are planned and delivered in short, fixed-length time slots, also known as sprints or iterations [23]. Application requirements are established and refined through active user involvement within the software development process. In this case, external clinicians were recruited to fulfill the role of the user because of the sensitive nature of SafePlan’s target audience.

**Figure 1:**
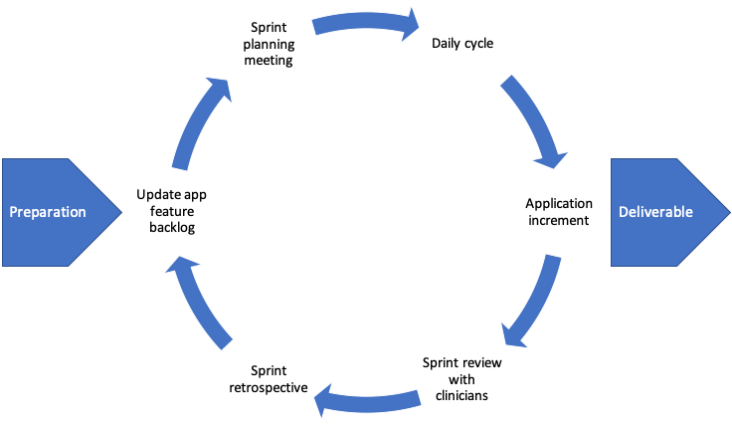
Design workshop process.

Once the initial app requirements were gathered, they were prioritized into a feature backlog based on their importance and completeness, that is, a proposed app feature was placed toward the bottom of the backlog if it was not yet fully defined. The sprints were typically 2-3 weeks in length and contained a number of stages (Figure 1). A full team review took place at the end of each sprint where the development team and external clinicians provided feedback on the tasks that had been carried out and any possible improvements that could be made. This effectively provided a form of continuous acceptance testing throughout the app’s development lifecycle.

### Requirements Elicitation

A survey (Multimedia Appendix 1) was circulated to frontline health care professionals to assess what features they would prioritize in a new mobile app relating to mental health and suicide prevention. The survey comprised a list of questions relating to the overall usefulness of mobile app technology to their service needs and consisted of 18 potential components that could be incorporated in the new app. There were 15 respondents to the survey, all health care professionals involved in the treatment of adults or children with mental health difficulties.

The majority of respondents were confident that mobile apps could be used in their service, for example, in session, between sessions, as an intervention itself, as an adjunct to other interventions, for younger clients, or as a tool for staff. All but 4 of the respondents used apps in their work or signposted/recommended particular apps to their clients. The most popular apps used and recommended were mindfulness-based apps, including Headspace and Smiling Mind [24,25].

There were concerns raised around using mobile technology to treat patients relating to confidentiality, accessibility, usability, and reliability. These concerns were noted and significantly informed about the app design process, as will be discussed in detail later. The respondents were also asked to independently rank the 18 potential app components in order of usefulness. The top 5 ranked features are set out below:

1. Interactive safety plan that can be recorded and potentially shared
2. Coping skills/training tools (eg, mindfulness exercises, DBT worksheets)
3. Tracking of symptoms/behaviors (eg, behavioral activation, urge to self-harm, suicidal thoughts, and coping skills used)—guided by DBT cards
4. Opportunity to link warning signs with specific coping skills to aid problem solving in times of distress (with a therapist)
5. Details of crisis support services/contacts

These features formed the basis for the initial app design. A number of features were deemed less important but still desirable, and as a result, they were placed on the *nice to have* list, that is, they would be implemented, time and resource permitting. These included components for tracking sleep, mood, and exercise, a capability to visually represent user data using graphs and allowing for a privacy function, that is, the app requires a log-in or passcode to gain access.

### Design Process

A design group was established to design and develop the app. The group consisted of 6 members and was multidisciplinary in nature with expertise in a variety of fields such as mHealth, computer science, clinical psychology, and suicide prevention.

In addition to this core group, the design process was supplemented with the inclusion of 5 additional health care workers who took part in the clinician survey. It was agreed that their input would be captured in the form of design workshops over the course of the app’s development lifecycle. This iterative method of having regular checkpoints with external clinicians proved to be a valuable way to ensure that the app’s construction was aligned with the initial requirements. The design workshops took place at the National University of Ireland, Galway (NUIG), and were held every 2 months during the SafePlan’s design and development phases.

Input and evaluation were provided through the design workshops, and this informed which app features were worked on for that particular development cycle. In an agile approach to software development, these features are formed through the process of refining *User Stories*. A user story is a short, simple description of a requirement told from the perspective of the person who desires the new capability, usually a user or customer of the system [26]. In the context of SafePlan, the user stories originated from the initial requirements survey and were refined over the course of the design workshops.

### User Interface Design

Before any development work was carried out, a significant amount of time was spent on creating a user interface (UI) design for SafePlan. Early prototype designs were developed based on the functionality gathered during the requirements elicitation process. These prototypes were designed in accordance with industry-standard design guidelines for mobile app development as well as being informed by a human-computer interaction expert on the design team (with extensive experience of UI design in both industry and academia). Using JustInMind [27], a tool for creating interactive wireframes, a variety of screen mockups were presented during the first design workshop where a number of adjustments were suggested by the attending clinicians based on their experience of working with patients in the area of suicide prevention. These suggestions were mainly focused on usability and resulted in minor updates to the app’s overall UI design. For instance, the initial mockups used individual buttons to capture a user’s feelings or urges within the DBT diary section of the app. The clinicians were all in agreement that this would be better presented in the form of a slider scale, as this approach should be more familiar to potential users based on their experience with similar apps.

**Figure 2:**
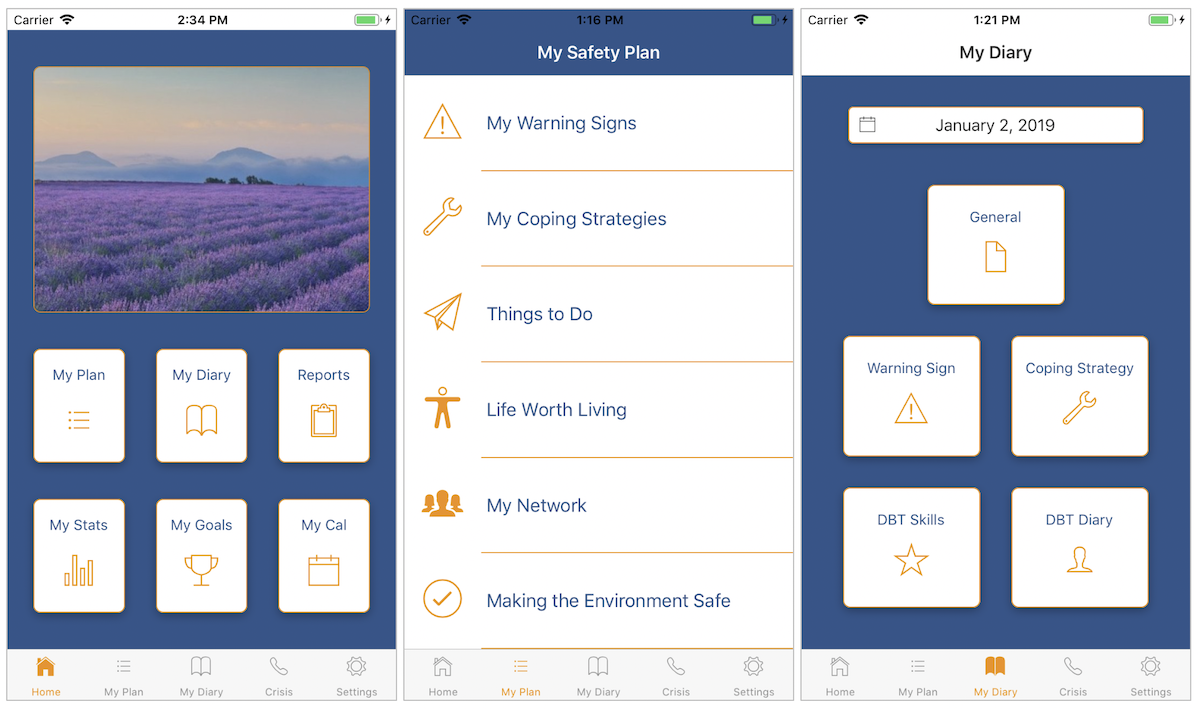
Screenshots from Home, Plan and Diary tabs within the SafePlan app.

Owing to the vulnerable nature of SafePlan’s intended users, it was of extreme importance that the app’s content was presented in a simple and well-structured manner. The design team felt it was crucial that navigation to the app’s 2 core components—Safety Plan (*My Plan*) and Diary (*My Diary*)—was always evident to the user no matter where they were located in the app’s screen hierarchy. This is why both components can be accessed through shortcut buttons on the home screen and via the bottom tab bar, which remains visible on every screen within the app. This can be seen in the screenshots that display the app’s Home, Plan, and Diary UI (Figure 2). Care was also taken to ensure that the pictorial imagery available was of a sensitive nature.

The final step in completing SafePlan’s visual design was devising a color scheme for the app’s UI. Following a presentation of alternate themes, the design team decided on a complementary color scheme for SafePlan. A complementary color scheme uses one base color and its complement, the color on the exact opposite side of the color wheel [28] as an accent color to draw attention to important elements on the screen. A base color of deep blue was chosen by the design team, as blue is a calming color, with the associated accent color being orange. Other color schemes, such as monochromatic and analogous, were explored in earlier design workshops; however, a consensus was reached to proceed with a complementary approach.

### Use Cases

**Figure 3:**
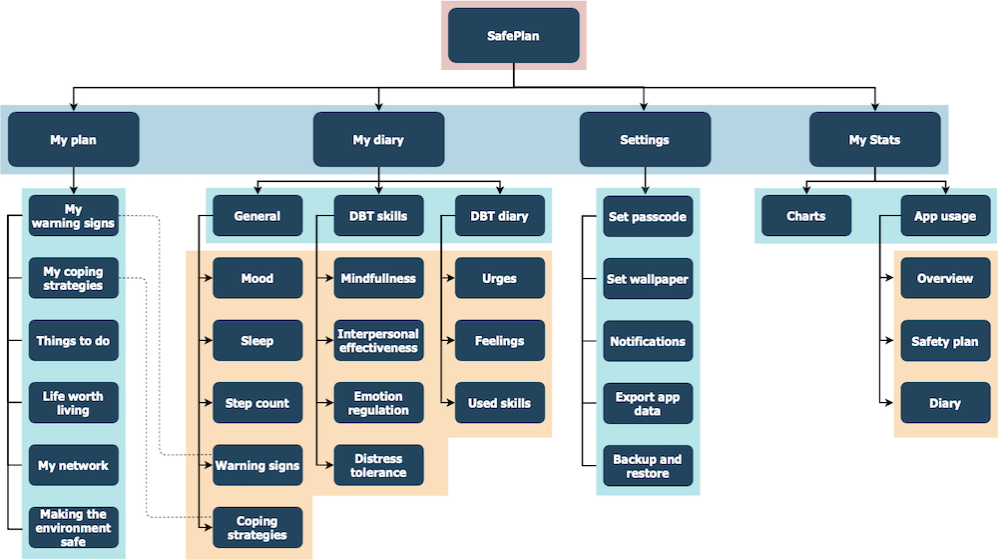
Functional design of SafePlan use cases.

A functional design structure chart (Figure 3) was created based on the requirements feedback obtained from the clinician survey. This outlines the overall depth of the app’s wide variety of use cases that are spread across its core components. These use cases were approved at the first design workshop, and a development schedule was prepared to identify the priority of each app component and the timeline expected to complete each one.

#### Safety Plan

As discussed, the highest ranked and most requested feature for the app was a shareable interactive safety plan. The safety plan was based on the text-based Safety Plan Template by Brown and Stanley [29], and was digitally enhanced to enable attaching of relevant media to the various sections within the plan, that is, images and videos could be associated with a user’s particular coping strategy.

It was also suggested that a user should be able to link their warning signs with specific coping strategies to help with problem solving in times of distress. This feature was added via the *New Warning Sign* screen, where the user can select appropriate strategies from a list of their saved coping strategies within the app. This is a crucial attribute in any safety plan; the user can address a particular warning sign before it fully manifests by following the applicable linked coping strategies. This highlights another key benefit in maintaining a user’s safety plan in digital format, as this type of automatic linking could not be achieved using a paper-based approach.

#### Diary

The diary element of the app is divided into 2 distinct parts. The first is a general diary where a user tracks their overall mood, sleep, or any thoughts they want to note for that day. The second centers around DBT, a well-researched treatment program that aims to help people who have difficulties managing intense emotions [30]. All health care workers participating in the focus group were in favor of including an interactive version of a DBT diary card within the app. This involves the user entering a rating, generally between 0 and 5, for specific feelings and urges they experience throughout the day. It would also enable the user to track what DBT skills they have used over a certain time frame.

One of the key objectives behind the development of the SafePlan was to ensure that it provided access to mental health support for as many people as possible. Appropriately, the DBT option was added to the app’s settings menu, whereby a user could activate/deactivate the DBT material within the app. This was to safeguard against users with no experience of DBT treatment feeling overwhelmed or that the app would not be suitable for them. When the DBT option is switched off, the diary component is still a valuable tool as the user can track mood, sleep, and save any warning signs or coping strategies they have encountered during that day. The DBT option is switched off as a default setting when initially downloaded from the respective app stores.

It was also felt that this customization could provide a unique feature for future versions of the SafePlan. The ability to enable or disable the DBT material allows for the potential to incorporate further treatments within the app. For instance, following the same format, other therapeutic interventions could be integrated into a newer version with the same activation functionality, for example, cognitive behavioral therapy (CBT) for suicide prevention. Facilitating this type of tailored approach to mental health treatments could possibly expand the app’s user base even further.

#### Clinician Reports

Eliminating the need for patients to maintain paper plans and worksheets was an important motivation for developing the app. This was a common challenge reported by clinicians involved throughout the focus group sessions. They explained how easy it is for paper reports to get lost or damaged between weekly sessions and that this can lead to a lack of consistency in patient data. Informed with this knowledge, the design group invested significant effort in establishing the correct format in which to summarize the safety plan and DBT data so that they could be used by clinicians and be available as a replacement for paper reports.

It was agreed that the report structures would remain similar to their paper counterparts. The safety plan report is a point-in-time PDF document that compiles all the entries in the various plan sections. The DBT diary card report is slightly different in that the user can select the specific seven-day period in which they would like to view their entries. This was to ensure that consistency was maintained between traditional DBT diary cards, which are generally updated on a weekly basis.

The aggregation of a user’s digital data and its presentation proved challenging for the design team. The collected data relates both to the user’s *diary*, stored in the app’s local database, and their overall app usage. It was agreed that this information would be most effective in chart form. The user can access the diary charts through the *My Stats* section of the app. The charts are dynamic in that the following options can be selected:

1. Diary item to analyze
2. Period (eg, past 7 days, past 30 days)
3. Diary item to compare with (not applicable with DBT skills)

Tracking a user’s overall usage of the app was identified as a potential key tool for clinicians during the second focus group session. Being able to know when a user has viewed a certain safety plan item or even how many times an item has been viewed could provide key insights to not only the therapist or clinician but also the user themselves. Similar to the diary data, these insights can be viewed through the *My Stats* section of the app. They are predominantly centered around the safety plan inputs and are divided into 3 types (last entered, last viewed, and most viewed) for each plan section, that is, warning signs, coping strategies, and contact.

One final method for data analysis that was incorporated into the app is the facility to export diary and usage data as a comma-separated values (CSV) file. This was requested by the clinicians, as it enables further analysis of the CSV data, using statistical software, into long-term trends that are captured through the various diary inputs and usage patterns of their patients. It could also support cohort analysis of patients to identify associations between variables. From these data, a clinician might, for example, observe that a patient’s overall mood is significantly affected by either their quality of sleep or one of the many DBT diary card urges/feelings, such as suicide, self-hand, and anger. Patients could then work with their clinicians to identify the necessary steps to take to help manage their mood more effectively.

In collaboration with their clinician, the user is invited to set up regular notifications to remind them to (1) record agreed-upon behaviors and (2) share their data. This feature is included to enhance adherence. Importantly, and following considerable stakeholder input with clinicians, the user will share their data in person with their clinician. User data will not automatically be shared and will not be monitored outside of these agreed-upon times, and this will be made clear to users upon app download. In addition, a copy of the Safety Plan created in-session (to include who will support the person in times of crisis and how) will be shared with the named individuals identified in the plan to ensure that the safety plan is adhered to and supported. To ensure that data are not lost if a user changes the device, for example, users are reminded to back up their data regularly.

The method for sharing CSV files is discussed in the following section.

### Technologies Used

The app was built using React Native, a framework for building native mobile apps for iOS and Android devices [31]. It enables the development of native apps using JavaScript and hence greatly reduces implementation time, as two codebases do not have to be maintained when building an app for both platforms.

Owing to the confidentiality and security concerns that were highlighted as part of the clinician survey, it was decided that the app’s data should be stored on a device’s local database rather than being uploaded to external servers. SQLite was used for the local database because it is lightweight, fast, and reliable [32].

Sharing and exporting data from the app also impacts privacy and security-related concerns raised by the clinicians. In order for the app to be effective, it is essential that a user shares their safety plan or weekly diary card with their clinician in a secure manner. This requires a thorough analysis, and many sharing strategies are considered. A detailed review (Table 2) was carried out to establish what approaches similar apps (mobile apps used as an adjunct to therapy) had used to extract user data for an external party, that is, a clinician. The review examined a wide variety of methods that have been used previously to share user data between mHealth apps and invested third parties, that is, clinicians. The majority of apps reviewed were using some form of an external server or cloud portal where the user could upload their data for a clinician to access. This sharing technique requires additional security resources because of the handling of sensitive user data and, consequently, may result in further legal and ethical considerations. A different approach, adopted by CBT-I Coach, provided functionality, whereby the user could email their sleep data to themselves so that they could print it and share with their clinician.

**Table 2:** Review of existing apps’ sharing strategies.

References	App names	Sharing methods
Kuhn et al [33]	CBT-I Coach	Users can email data to themselves
Matthews and Doherty [34]	Mobile Mood Diary	Users asked if they would like to upload their data from the app to an external server. A web-based tool allows users to visualize their recorded data. Only users have log-in credentials for this tool, and the idea was that they would log on at the beginning of their session with their therapist so they could review
Rivzi et al [35]	DBT Coach	Button within app’s settings menu that a user could press to send data. Data would be uploaded to a cloud file that could be accessed by research staff
Reger et al [36]	PE Coach	App did not facilitate electronic sharing of any data. This was noted as a limitation of the app by the researchers
Pramana et al [37]	SmartCAT	Therapist can access user’s data through a web-based portal

Having conducted the review and discussed the potential implications of each strategy with the app stakeholders, a safety-first approach was adopted to extract user data. The design group concluded that all the control should remain with the user and decided against the facility to upload data to any cloud file or external server. In the current version of the app, a user can generate a report of either their safety plan or DBT diary card for a specified period. These reports can then be printed directly from the user’s device or can be saved as a PDF file and distributed as they themselves might require. This solution satisfied the criteria for ensuring that the user is fully responsible for their data and that it will stay within the user’s environment unless they decide otherwise.

### Implementation and Testing

Following the design workshops and development sprints, the first version was implemented for beta testing. The app was tested internally by the core design team, after which final changes were made to remove bugs and prepare the app for external interaction.

Ethical approval was granted by the School of Psychology Ethics Committee at the NUIG on May 05, 2016, to evaluate the Corofin Abbeyknockmoy, Athenry, Turloughmore Community Health (CAATCH) app. This app was also developed by the design group to raise awareness and provide information about local support services. Ethical approval was granted by the School of Psychology Ethics Committee NUI Galway on May 28, 2019, with respect to the SafePlan study.

On completion of beta testing, students (N=18) were recruited from a local secondary school for an evaluation session. The group was representative of age (14-16 years) and technology experience (they used their own mobile phones, both Android and iOS, with the devices and user technology experience being representative of this demographic) of one of the target user populations. Their role was to test the usability and functionality of the app, rather than the actual content. The students were carefully screened in advance of participating in the testing process, via a consent process involving parents (Multimedia Appendix 2), and clinicians were available onsite during the evaluation session to answer any questions. The need for representative users was required due to the sensitive and vulnerable nature of SafePlan’s intended audience. In addition, any interaction with this audience would require intervention by clinicians over a longer period. There are plans for this type of testing in the next stage of the SafePlan project through the form of a randomized controlled trial (RCT).

The students were asked for their opinion on the overall functionality and usability of the app. A proposed task list was created (Multimedia Appendix 3) to ensure that coverage of the app’s features mirrored its expected long-term normal usage. This is a typical approach adopted for the usability testing of mHealth apps [1]. After the students had carried out these tasks, they were asked to complete a system usability survey (SUS) [38], a well-known tool in usability practice and research, and an open-ended evaluation questionnaire. The SUS is an industry-standard 10-item scale that explores the perceived usability of a technological application. The scale has excellent psychometric properties with measures of reliability scoring over 0.90, good indicators of validity, and sensitivity [39]; and a Cronbach alpha of .92 [38]. Norming studies [39] have presented normative data providing an empirical basis for the interpretation of SUS scores. According to such normative data, an SUS score of 65 can be interpreted as a marginally acceptable result. More recently, Finstad [40] compared the original five-point Likert scale instrument with a modified seven-point Likert scale version. The findings supported the conclusion that seven-point Likert items provide a more accurate measure of a participant’s true evaluation, and therefore, this was the approach adopted in this study.

## Results

### Quantitative Analysis

The quantitative data determined from the SUS are presented in Multimedia Appendix 4. Participants recorded higher scores for ease of use and confidence in using the app. There was a relatively high mean score (3.17) in the last question on the SUS with regard to users needing to learn many things before commencing using the app. However, this question also resulted in the highest SD (1.95), suggesting that a greater spread of ratings occurred for this question.

The question relating to inconsistency within the app received the lowest mean score (2.28) and the lowest SD (0.89). These markers indicate that the participants came to a consensus that the app provides users with an overall cohesive experience. The mean overall SUS rating was 71.85 (SD 1.38). An SUS score above 68 is considered above average based on research in this area [41]. Although the SafePlan scored well in this regard, the usability testing was carried out by representative users, and therefore, the app’s functionality was not entirely relevant to them. This score is likely to increase when tested with real users.

### Qualitative Analysis

The feedback collected through the evaluation questionnaire is categorized and summarized below. This was carried out following a thematic analysis approach. Thematic analysis is a method for identifying, analyzing, and reporting patterns within data [42].

#### Theme 1: UI Design

The SafePlan design and UI were well received by the majority of participants. The intuitive structure and overall simplicity of the visual appearance were frequently noted in the questionnaire responses:

I like the set up. It’s visually pleasing and easy to read. Planning was somewhat fun!

P4

Simple easy UI. Many different uses for all users and cases.

P5

The shade of orange used in the color scheme was disliked by two of the participants. They noted that this color was quite sharp and may potentially unsettle users. However, this particular participant also remarked that the app’s primary color of blue worked well because of its calming nature:

I didn’t like the colour orange on the apps cover. The blue was a good idea because it’s a relaxing colour but the orange is intense.

P10

Two more participants commented on the switch interface used to change the app’s home screen wallpaper. During the session, they suggested that this would be more intuitive if it was implemented with a button instead:

...the fact that the set wallpaper has a slider.

P5

#### Theme 2: SafePlan Tutorial

Although the overall UI was rated highly by the participants, it was suggested that adding some form of tutorial for first-time users would be helpful. This could possibly be in the form of a video directly linked within the app or, alternatively, a number of nonintrusive dialog boxes could appear on initial downloads containing instructions for key components within the app:

...quick tutorial the first time you enter each screen (speech bubbles of important points).

P5

Some features were initially a bit difficult to locate and understand but other than that it was excellent (tutorial would be helpful).

P9

Similarly, the high number of functions available within the app proved to be overwhelming and difficult to locate for some participants during the evaluation session:

Not everything is completely obvious with how to use.

P17

However, there was a sense that once they got the required directions and after some time had elapsed, the app’s value became more apparent:

It is a bit confusing to get into the flow of the app, but you can understand it quickly.

P16

#### Theme 3: Confidentiality

At the beginning of the evaluation session, the participants attended a presentation that detailed the background and development of the app. The storage of user data created within the app was explained, and this was positively noted by one participant in the questionnaire:

I liked how the information was strictly confidential. I feel it will allow the user to be more at ease using the app.

P8

The ability to secure the app with a passcode was also remarked upon favorably by the participants. They felt this was a fundamental feature for the app because of the nature of the information being recorded and stored:

I liked how organised and accessible the app was. I also thought that the way you could put a passcode on the app was important.

P11

#### Theme 4: Additional Functionality

The final point in the evaluation questionnaire asked the participants if they would like to see any additional features incorporated into the app. This is a valuable open-ended question, as some of these suggestions may influence future versions of the app.

The idea of a food tracker diary was mentioned by more than one participant. A place where a user could record the food they have eaten for a particular day and obtain insights based on the data collected over a specified period of time. This would be a natural supplement to the app’s diary component, where the user already has the ability to record their mood, sleep, and step count on a given day:

...maybe a food tracker because some people may not believe they are eating too much / too little when they are.

P9

Another recommendation from one of the participants focused on providing the app user with motivational messages each morning through the form of push notifications:

Maybe some relaxing videos or a notification each morning with something motivational.

P1

Finally, adding calming music or sounds to the app was also quite a popular suggestion. The following participant brought this idea up in connection with aiding the user to fall asleep. This feature could potentially be linked with the app’s sleep diary in future versions of the app:

You should add a feature where you can add in calming music or noises to fall asleep.

P10

## Discussion

### Principal Findings

The principal findings of this project were obtained from acceptance and usability tests, which were based on feedback from health care workers and representative users. Health care workers were recruited to provide iterative feedback on the app’s design and functionality throughout its development lifecycle. This feedback was key to finalizing the app from a clinical perspective, as it ensured that no unnecessary or inappropriate features were added to the app’s portfolio. Usability testing was undertaken with representative users in terms of age (14-16 years) and technology experience. Representative users were necessary at this early stage of testing because of the sensitive nature of SafePlan’s intended audience. Carefully screened students from a local secondary school were invited to attend an evaluation session, where they were presented with a number of specific tasks to carry out on the app to determine its overall usability.

The feedback received from the usability testing day was largely positive. The participants perceived the main benefits of the SafePlan app were its overall UI design and emphasis on user confidentiality. In particular, the acknowledgment of the app’s privacy features was a strong vindication for the design team, as this was an area of significant focus during the initial planning meetings. The most common issues in mHealth are privacy and data security [43]. These concerns were addressed from the outset of SafePlan’s development by ensuring that all user data are stored locally on their own device and providing the user with an option for securing the app with a passcode.

Although the majority of evaluations were positive, a small number of potential improvements were identified for future versions of the app. The amount of information content and functionality within the app was somewhat overwhelming for some participants on initial use. There were suggestions that an introductory tutorial video for first-time users could be extremely beneficial to give them a general overview of the app’s features and use cases. On this point, it is also worth noting that the participants’ experience with SafePlan was not representative of real, ongoing use. It is expected that the majority of the app’s users will be guided through its content by a clinician, and this may mitigate any confusion they may experience on initial use. Another participant proposed that the app provides users with motivational messages or relaxing videos each morning through the form of push notifications.

### Limitations

Although the usability and acceptance testing proved to be beneficial in designing and developing a clinically informed, user-friendly mobile app, further ethically conducted evaluations involving patients with suicidal thoughts or behavior will provide much greater insight into whether participants interact with the app in the way it was intended. Previous researchers have highlighted the need for more robust testing of mHealth apps within clinical settings [44]. The research team has established experience in co-design, iterative design processes, and the selection and use of measures to evaluate user engagement and digital intervention usability [45-47]. A pilot RCT investigating the feasibility of using SafePlan as an adjunct to therapy in mental health services is currently being planned (HRB CSF 2020–010). The aims of this trial are to (1) assess the feasibility and acceptability of the SafePlan intervention as an adjunct to therapy for individuals at risk of suicide accessing Irish mental health services, (2) examine the feasibility of a definitive RCT of the SafePlan intervention, and (3) assess the feasibility and acceptability of the SafePlan data collection methods (including in-built ecological momentary assessment) for clinicians and patients.

This is particularly relevant when it comes to observing a user’s long-term interaction with the app’s various diary and safety plan functions. It is anticipated that a user will maintain and update their data within the app on a regular basis, and this is where the most value will be gained, both for the user and their clinician. The long-term usage of health apps is highly significant, as some studies have reported log-ins dropping sharply to nearly zero after 1 month from download, even for the highest rated apps [18]. With this in mind, further recording of user feedback during the pilot stage is required to determine if the app has the desired lasting effect on its users, and this would be a key element of a follow-on study with end users. Currently, there is no warning on the app in terms of usage, and a cautionary preamble section will be added to the app stating that it is only to be used after undergoing an orientation session. Furthermore, currently, there are no automated warning notifications built into the system, and this has been identified as a future requirement, whereby patients will be informed when they should consider seeking professional help.

### Comparison With Prior Work

Many members of SafePlan’s core design team were previously involved in the development of another app relating to improving outcomes in suicide prevention. The CAATCH app [44] was released in 2014, and its main goal was to raise awareness, provide information, and signpost to education and training programs available at the community level within the local area. In terms of the involvement of end users, CAATCH involved members of the public and patients in the design and testing phases, which guided the overall intervention and subsequently shaped the design of the current SafePlan app. It was the experience of young people accessing mental health services and actively seeking a digital means of storing and using their SafetyPlan/DBT code cards, which ultimately led to the development of the SafePlan.

Although SafePlan also aims to raise awareness and provide information, it operates at a patient-clinician level where it can be used as an adjunct to treatment. It is a significantly more interactive app in that it accepts various forms of user input and provides tools for visualizing this input in the form of charts and reports, so that users can analyze their data over a specified period.

### Conclusions

In conclusion, through the process of iterative design with the input of targeted clinicians and key design personnel, a fully functional version of the SafePlan mobile app was developed. This app has the potential to offer an accessible adjunct to face-to-face therapy and to support the generalization of skills acquired in-session to beyond the clinical setting. It also provides useful functionality that may lead to improvements in the interaction between a patient and their clinician. This can primarily be demonstrated through the process of transferring previously recorded paper-based reports and worksheets to specific sections within the app. This facilitates more accurate and timely data recording practices and subsequent analysis, with a correspondingly positive impact on treatment outcomes.

Following on from our review of existing mobile apps providing support in the area of mental health and suicide prevention (as presented in Table 1), further apps have been added to the app stores [48]. With approximately 20,000 mental health apps now reported in the app stores by the American Psychological Association [49], this number will continue to grow, whereas the core challenge of maintaining privacy, providing reporting tools for controlled sharing of user data, and combining safety planning with other therapeutic interventions remains. Safety planning intervention is an evidence-based brief intervention for suicidality, which is increasingly considered to be the best practice in terms of health care practice [6]. SafePlan potentially operationalizes a more interactive and individualized means of implementing this intervention while also offering the potential to combine this safety planning intervention with other prevention techniques (eg, DBT or CBT) in one flexible app. This intervention flexibility was welcomed by all participating clinicians, as it provides the potential to open the app to a wider user cohort.

SafePlan will require further evaluation from users within the target population. This was a topic of consideration for the design team, and it was agreed that because of the nature of the app’s intended users, it would not be appropriate to involve patients at this early stage of development. The combination of input from clinicians and groups that were partially representative of the end users was seen as a valid substitute for patients’ evaluation until sufficient evidence was gathered to carry out a pilot implementation with the app’s intended audience, for example, a pilot study with patients and clinicians to gain further insight.

## Appendix

Multimedia Appendix 1 List of eighteen potential app components used in the initial app survey:.

Multimedia Appendix 2 Evaluating the SafePlan App Parent/Guardian Consent Form.

Multimedia Appendix 3 Task list for usability testing day.

Multimedia Appendix 4 Responses were scored on a 7-point Likert scale ranging from 1=strongly disagree to 7=strongly agree.

## Abbreviations

CAATCH: Corofin, Abbeyknockmoy, Athenry, Turloughmore Community Health
CBT: cognitive behavioral therapy
CSV: comma-separated values
DBT: dialectical behavior therapy
HSE: Health Service Executive
mHealth: mobile health
NUIG: National University of Ireland, Galway
RCT: randomized controlled trial
SA: suicide attempt
SUS: system usability scale
UI: user interface
WHO: World Health Organization
