# Supplementary material for: A Mobile Health Approach for Improving Outcomes in Suicide Prevention (SafePlan)
Source: J Med Internet Res. 2020 Jul 30;22(7):e17481. doi: 10.2196/17481 (PMC7426795; doi:10.2196/17481)
Supplement: Multimedia Appendix 1 [file jmir_v22i7e17481_app1.docx]

## Appendix 1

*List of eighteen potential app components used in the initial app survey:*

- Interactive safety plan that can be recorded and potentially shared
- Tracking of symptoms/behaviours (e.g., behavioural activation, urge to self-harm...)
- Tracking of related risk factors (e.g., sleep, medication adherence, mood…)
- Capability to share data (e.g., in session)
- Coping skills/Training tools (e.g., mindfulness exercises, challenging NATs, DBT worksheets)
- A feature where the individual can identify their warning signs
- Privacy function - app is login or password protected
- Reminder functions for appointments/medication use
- App to be an adjunct to therapy, used by individuals already accessing mental health services
- Monitoring/Traffic light system that will alert the user when warning signs are apparent
- Opportunity to link warning signs with specific coping skills to aid problem-solving
- Timestamping of information collected (to support identification of triggers in session)
- App to monitor the use of the safety plan
- Evaluative function (e.g., user ratings, data collection on app use)
- Capability to visually represent client data (e.g., graphs)
- Suicide risk assessment measures available to complete on app (e.g., C-SSRS, BDI, BSIS)
- Details of crisis support services/contacts
- Opportunity for the individual to identify and record contact details of their support network
