# Supplementary material for: A Mobile Health Approach for Improving Outcomes in Suicide Prevention (SafePlan)
Source: J Med Internet Res. 2020 Jul 30;22(7):e17481. doi: 10.2196/17481 (PMC7426795; doi:10.2196/17481)
Supplement: Multimedia Appendix 2 [file jmir_v22i7e17481_app2.docx]

## Appendix 2

*Evaluating the SafePlan App*

**Parent/Guardian Consent Form**

The School of Psychology and the School of Computer Science in NUI Galway, along with clinicians and health professionals in the HSE, have developed a mobile phone app called ***SafePlan*** to support a key national mental health initiative to reduce suicidal behaviour. The app is aimed at young people where we know there is a widespread smartphone ownership.

**SafePlan** is under preliminary field-testing trials and user evaluation. We are seeking the assistance of TY students in XXXXXX to provide general feedback and help us test the usability and functionality of the app, rather than the actual content. The content of the app has been developed through extensive collaboration and consultation with various professionals and clinicians in the HSE. Participation in the ***SafePlan*** evaluation exercise will give TY students the opportunity to experience how smartphone apps are developed and tested prior to release on the iOS and Android platforms. During the evaluation session, we will have clinical psychologists and software engineers present who will provide a briefing on their respective roles on developing the ***SafePlan*** app.

When we have completed our content design and technical development of the app it will be promoted for widespread use by health professionals and their clients.

The evaluation session will be held on **Monday, XXXXX** at **9.30am in Room G065** in the **XXXXX**. The session will conclude around 12.30pm and we will provide lunch vouchers for all participating students.

This evaluation project has been approved by the School of Psychology Ethics Committee but because of the subject matter associated with the app, we wish to bring to your attention, as parents/guardians, the following:

- Some of the content contained in the app is of a sensitive or emotional nature
- If your son or daughter has been affected by suicide or any other loss in the past 12 months, it may be advisable not to attend
- Clinicians will be on-site during the evaluation session to support your son or daughter
- If you have any concerns about your son or daughter’s mental health, please contact your GP or see [www.yourmentalhealth.ie](http://www.yourmentalhealth.ie) for advice, information and details of local support services.

Thank you,

---------------------------------------------------------------------------------------------------------------------------

I have read this information sheet and I grant permission for my son or daughter to take part in this event:

Student’s Name: --------------------------------------------------------------------

Signature of Parent/Guardian: --------------------------------------------------------------------

Date: --------------------------------------------------------------

**
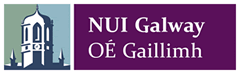
**

**TY STUDENT CONSENT FORM**

**Please initial box**

1. I confirm that I have read the information sheet for the above. □

study and have had the opportunity to ask questions.

2. I am satisfied that I understand the information provided and. □

have had enough time to consider the information.

3. I understand that my participation is voluntary and that I am. □

free to withdraw at any time, without giving any reason.

4. I agree to take part in the above study. □

Name of Student Date Signature
