# Supplementary material for: A Mobile Health Approach for Improving Outcomes in Suicide Prevention (SafePlan)
Source: J Med Internet Res. 2020 Jul 30;22(7):e17481. doi: 10.2196/17481 (PMC7426795; doi:10.2196/17481)
Supplement: Multimedia Appendix 3 [file jmir_v22i7e17481_app3.docx]

## Appendix 3

*Task list for usability testing day:*

**
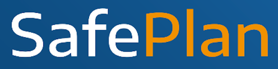
**

Please complete the following tasks using the ***SafePlan*** mobile application. You may ask any of the facilitators present questions in order to help you complete the tasks.

**My Plan Tasks:**

1. Go to *MyPlan* section of the app.
2. Go to *My Warning Signs*. Add a new *Warning* *Sign* “I have trouble sleeping or I sleep too much”.
3. Go to *Things to Do*. Add “Go for a Walk with Dog” (you can insert your actual dog’s name here, e.g. *Snoopy*) to *My Things.*
4. Find a helpful Distraction from the list and write it down here:

___________________________________________________

1. Add a person from your phone contacts to *My Network* and designate them to be a “Helper”. Under *MyReports/Safety Plan*, verify that they have been added ok.

**MyDiary Tasks**

1. Go to *MyDiary* section of the app.
2. Add a diary entry under *Warning Sign* “I have trouble sleeping or I sleep too much” and confirm that it has been entered by checking the Safety Plan on *MyStats / App* *Usage*.
3. For the past five days (i.e. Thurs Feb. 7^th^ to Mon Feb. 11^th^), enter the following data, and check the resulting graphs under *MyStats/Charts.*

| **Day** | **Mood Scale** | **Sleep Scale** | **Step Count** |
| --- | --- | --- | --- |
| 1 | 2 | 5 | 7000 |
| 2 | 3 | 4 | 3000 |
| 3 | 4 | 3 | 13000 |
| 4 | 5 | 4 | 4000 |
| 5 | 4 | 5 | 7000 |

**MyGoals Tasks:**

1. Go to *MyGoals* section of the app.
2. Add a new goal (steps) with a target of 10000. Check this goal on a graph under *MyStats/Charts*.
3. Add a new goal for Mood Scale: Target Rating 4 and call it “Good Mood Goal”.

**MyCal Tasks:**

1. Go to *MyCal* section of the app.
2. Add an appointment with your Doctor for February 15, 2019 from 11h30 to 12h00.

**Editing Tasks:**

1. Change the photo on the home screen (Wallpaper).
2. Share the overview of *MyStats/AppUsage* via an email to yourself.
3. Change your saved Doctor’s appointment from 11h30 to 09h30.
