# Supplementary material for: A Mobile Health Approach for Improving Outcomes in Suicide Prevention (SafePlan)
Source: J Med Internet Res. 2020 Jul 30;22(7):e17481. doi: 10.2196/17481 (PMC7426795; doi:10.2196/17481)
Supplement: Multimedia Appendix 4 [file jmir_v22i7e17481_app4.docx]

## Appendix 4

**Responses were scored on a 7-point Likert scale ranging from 1=strongly disagree to 7=strongly agree.*

| **Question*** | **Participant mean** | **Participant median** | **Participant SD** |
| --- | --- | --- | --- |
| I think that I would like to use the SafePlan app frequently | 4.67 | 5.00 | 1.88 |
| I found the SafePlan app to be unnecessarily complex | 2.67 | 2.00 | 1.46 |
| I thought the SafePlan was easy to use | 5.39 | 5.50 | 0.92 |
| I think I would need the support of a technical person to be able to use the SafePlan app | 2.78 | 2.00 | 1.80 |
| I found that the various pages in the SafePlan app were well integrated | 5.56 | 6.00 | 0.92 |
| I thought there was too much inconsistency in the SafePlan app | 2.28 | 2.00 | 0.89 |
| I would imagine that most people would learn to use the SafePlan app very quickly | 6.00 | 6.00 | 1.08 |
| I found the SafePlan app very awkward to use | 2.61 | 2.00 | 1.72 |
| I felt very confident using the SafePlan app | 5.00 | 5.00 | 1.24 |
| I needed to learn a lot of things before I could get going with the SafePlan app | 3.17 | 3.00 | 1.95 |

Table 3 Means and standard deviation for the SUS across the student testing group
